# Virtual Reality 360-Degree Films for Objective Structured Clinical Examination Preparation: A Descriptive Study

**DOI:** 10.7759/cureus.78120

**Published:** 2025-01-28

**Authors:** Ansam Khan, Vanessa Rodwell, Leya Luhar, Shakthipriya Nandakumar, Sharvez Sivam, Joell Rosil, Terese Bird

**Affiliations:** 1 Leicester Medical School, University of Leicester, Leicester, GBR; 2 Department of Medical Education, North West Anglia NHS Foundation Trust, Huntingdon, GBR; 3 Leicester Medical School, Ulverscroft Eye Unit, College of Life Sciences, School of Psychology and Vision Sciences, University of Leicester, Leicester, GBR

**Keywords:** 360, 360-degree, medical students, objective structured clinical examination (osce), osce, osces, pre-clinical, undergraduate, virtual reality (vr), vr

## Abstract

Background

Objective Structured Clinical Examinations (OSCEs) are a crucial part of medical school assessments, evaluating core clinical skills such as history-taking and communication. As medical school cohorts grow, delivering these resource-intensive examinations becomes increasingly challenging for educators. Students are known to have anxiety when facing OSCEs. This can be lessened with increased bedside teaching and clinical practice opportunities, which can be limited for students in the pre-clinical phase of medical school. Technology-enhanced learning (TEL), including affordable 360-degree video simulations, offers a viable alternative to face-to-face training. This study aims to demonstrate the non-inferiority of 360-degree resources compared to traditional teaching for OSCE preparation, providing an effective independent study tool.

Methods

Pre-clinical medical students were recruited (n=16) and randomly assigned to the virtual reality (VR) or control group. The virtual reality group utilised 360-degree videos. The control group received a traditional PowerPoint for OSCE preparation. Pre- and post-intervention questionnaires measuring self-perceived confidence and anxiety were completed by both groups. Students in the virtual reality group participated in focus groups to share their experiences.

Results

There was a statistically significant increase in confidence for OSCEs in both groups (n=12), demonstrating the non-inferiority of the virtual reality resource. The virtual reality group showed a significant increase in self-reported confidence (p=0.0117), and although their anxiety decreased, this was not statistically significant (p=0.1019). Thematic analysis highlighted the benefit of video in reducing anxiety and creating an immersive learning environment.

Conclusion

VR resources such as 360-degree videos show promise as a tool to prepare pre-clinical medical students for OSCEs, improving confidence and preparedness whilst reducing anxiety. The resource is especially promising in improving the diversity of patient cases. Whilst there is room for improvement in optimising the VR experience and expanding access, the findings support the inclusion of immersive technologies in medical education.

## Introduction

The UK's ageing population, coupled with rising pressures on the NHS workforce, has led to increasing demand for new doctors. The Medical Schools Council and the UK Government's NHS Long Term Workforce Plan aim to address this shortage by expanding medical school intake by 5,000 additional students annually, with a target of nearly 48,000 more doctors by 2036 compared to 2018 levels [1]. This expansion will significantly increase the number of medical students, which in turn amplifies the challenges facing medical educators in delivering high-quality teaching to larger cohorts.

Whilst many aspects of the medical school curriculum can be scaled up to accommodate growing student numbers, some areas, particularly clinical skills training and assessments such as Objective Structured Clinical Examinations (OSCEs), remain challenging. OSCEs are integral to medical education, assessing core clinical competencies such as history-taking, physical examination and communication skills. Medical schools require many staff and resources to deliver multiple mock OSCEs and continued bedside teaching with personalised feedback to prepare students adequately, which will become increasingly challenging as cohorts increase in number.

Students have been found to have anxiety when approaching OSCEs. In one study, it was suggested that "88% (of students) found the OSCE intimidating and more stressful than other forms of assessment" [2]. Reasons for this could be due to the wide coverage of knowledge required, difficulty in practicing and requirement for senior feedback. For pre-clinical students who have not yet started clinical placements, opportunities to practice these essential skills are often more limited. Therefore, we believe that pre-clinical students would benefit from some sort of extra asynchronous learning intervention that they can utilise in their own time to develop their clinical competency when approaching OSCEs.

Due to the nature of the OSCE, mark schemes are generally domain-based rather than checklists, reflective of real clinical practice, which students can find difficult to interpret in their preparation. Whilst OSCEs are supposed to be a reflection of clinical competence, it is commonly understood that the scenarios are more stereotyped examples of illness compared to clinical practice, due to the time constraints and the simulated setting of the examination. For pre-clinical students, the opportunities to practice and receive personalised feedback are limited, thus incorporating examiner comments into a resource would allow them to understand the examiner's thought process.

To address the issue of students' anxiety regarding OSCEs and to improve opportunities for practice, technology-enhanced learning (TEL) has emerged as a promising solution, offering immersive learning tools such as virtual reality (VR) and other simulated environments. Whilst previous studies have compared VR simulations with face-to-face learning, many of these studies relied on expensive equipment and focused on more advanced medical students [3,4]. One study investigating the use of 360-degree videos (without expensive VR systems) found that it was a viable and effective alternative to face-to-face teaching in a clinical setting, although it primarily targeted senior medical students and did not specifically examine pre-clinical learners [5].

By using 360-degree videos, which can be experienced with affordable devices (such as smartphones or standard computer screens), we aimed to provide an innovative solution for pre-clinical students preparing for OSCEs. To address the challenge of difficulty in accessing frequent personalised feedback, this study aims to demonstrate the non-inferiority of edited 360-degree video resources compared to traditional PowerPoint teaching for OSCE preparation, providing an effective independent study tool. These findings will contribute to ongoing efforts to improve clinical competency and support the growing demands on healthcare training.

## Materials and methods

Study design

Participants were selected from volunteer pre-clinical first-year medical students at the University of Leicester. Our inclusion criteria included first-year undergraduate medical students with no previous degrees and no previous formal teaching on OSCE methods, specifically in the topics of gastrointestinal and vascular presentations. Recruitment was carried out via email invitations, and participants were enrolled after showing interest. A total of 16 first-year medical students from the University of Leicester were recruited for this study. Students were randomly allocated to either the virtual reality group (n=8) or the control group (n=8).

A power analysis was conducted to determine the sample size needed to detect a significant effect of virtual reality (VR) on medical students' confidence scores. Based on prior similar studies, Cohen's d effect size was calculated as 1.33, suggesting a substantial improvement in confidence and indicating that a small sample size of seven students would suffice to achieve a power of 0.80, supporting this approach. Thus, our sample size of 12, after four students were lost to follow-up, was adequate to detect an effect. Furthermore, as this is a descriptive study rather than an experimental one, the goal was to demonstrate the non-inferiority of VR technology, rather than to test specific hypotheses. Our literature review showed that small sample sizes are common in descriptive research, particularly in single-institution studies with voluntary participants, where recruitment constraints often limit participant numbers [6,7].

The group allocated to the "virtual reality" arm accessed only 360-degree video resources without any accompanying live lectures. These 360-degree videos were the intervention, having been designed to simulate the OSCE experience, offering an immersive, virtual environment for students to practice and familiarise themselves with key OSCE scenarios. The videos were filmed using a 360-degree GoPro Omni camera, with student actors. Filmed scenes were authentic scenarios of clinical situations as would be used in OSCEs and edited with examiner comments explaining where marks were gained and lost, using University of Leicester year one mark scheme rubrics. These were then provided as asynchronous pre-learning materials on the University of Leicester's Blackboard platform. Students used Google Cardboard headsets to view the videos, or alternatively, the videos could be accessed via YouTube on a standard 2D screen. The VR group engaged with the videos independently in their own time before participating in a mock OSCE. Following the mock OSCE, a focus group was conducted with the VR group to explore their opinions on the use of 360-degree video as a learning tool.

The VR group then completed a pre- and post-questionnaires (Appendices) before and after watching two annotated 360-degree OSCE examination videos, consisting of a triadic consultation and a history-taking station. This provided a measure of any changes in their perception and preparedness for OSCEs.

However, the control group received small group face-to-face learning, designed and delivered by a trained OSCE assessor. They were given an in-person PowerPoint lecture on OSCE technique prior to the mock OSCE, covering the same content as the VR group, but in a live synchronous format. The VR group did not receive the live synchronous learning the control group did. The control group also participated in pre- and post-questionnaires.

Both the VR and control groups participated in a mock OSCE, consisting of a circuit of three OSCE stations to assess their history-taking skills. The mock OSCE stations consisted of a triadic consultation and two history-taking stations. Examiners were recruited via email to the local hospital trust and were all postgraduate doctors. All examiners had prior experience examining formative OSCEs at the University of Leicester. They were given a short examination crash course provided by an experienced OSCE assessor, to ensure the marking was standardised. The examiners were consistent across both the control and intervention groups and were blinded to the students' group assignments. As this study consisted of year one University of Leicester student volunteers, we avoided numerical scores and instead opted to use marksheets with Global Rating Scales (Fail, Borderline, Pass, Good and Excellent) with immediate verbal feedback, mirroring real year one OSCE formative assessments at Leicester.

The video resources used by the students are available to view in Videos 1-3.

**Video 1:** History-taking OSCE (dermatology) OSCE: Objective Structured Clinical Examination

**Video 2:** History-taking OSCE (headache) OSCE: Objective Structured Clinical Examination

**Video 3:** Triadic consultation OSCE OSCE: Objective Structured Clinical Examination

Data collection

Quantitative Data Collection

Quantitative data was collected using pre- and post-intervention (Appendices) self-rated questionnaires. A five-point Likert scale, ranging from "One - Not Confident" to "Five - Very Confident", was employed for each question to gauge changes in, for example, confidence levels, and anxiety, before and after the interventions. Examiners assessed students using Global Rating Scales with immediate verbal feedback.

Qualitative Data Collection

Qualitative data was collected through focus groups, conducted after the completion of VR intervention. We felt this was the best method as measures such as anxiety and preparedness for OSCEs are subjective. Participants were invited to reflect on their experiences with VR learning, including perceived strengths, challenges and its impact on their clinical knowledge and skill development. Each focus group was facilitated by an independent moderator, ensuring that discussions were unbiased and students felt comfortable sharing their experiences. Focus groups were audio-recorded and transcribed verbatim for analysis.

Data analysis

Statistical Analysis

For the pre- and post-session questionnaires, where the assumptions of normality and homogeneity of variance were met, paired t-tests were conducted to assess changes in self-perceived confidence and knowledge before and after the intervention. Statistical analysis was performed using GraphPad Prism 2024 version 10.2.0, with two-tailed chi-squared analysis applied to the Global Rating Scales data. A p-value of less than 0.05 was considered statistically significant for all tests.

Thematic Analysis

The transcripts were examined line-by-line and coded in Excel (Microsoft Corp., Redmond, WA) to identify significant concepts and ideas. Key sections of text were highlighted and systematically categorised into initial codes, which were further refined into sub-themes and grouped into overarching themes. These concepts were compared across both groups to identify emerging themes. Throughout the analysis, the researchers regularly compared their coding and thematic interpretations to ensure consistency, reliability and agreement on the identified themes.

Ethical considerations

Ethical approval for this study was obtained from the University of Leicester College of Life Sciences Ethical Approval Committee (approval number: 30173), and all participants provided informed consent prior to involvement in the study. Participants were informed that participation was voluntary and that they could withdraw from the study at any time without consequence. All data was anonymised to ensure confidentiality, and any identifying information was removed from transcripts and questionnaire responses prior to analysis.

All first-year medical students were given access to the VR videos on YouTube after completing the research, ensuring that there were no ethical issues regarding unequal access to the learning resources. It is important to note that students were provided access through university-supplied resources, including iPads distributed by Leicester Medical School, to ensure equal access to learning tools.

## Results

Quantitative results

Pre- and Post-intervention Questionnaires

The impact of 360-degree videos on first-year medical students' preparation for Objective Structured Clinical Examinations (OSCEs) was assessed through several key measures: confidence, anxiety, knowledge of OSCE procedures and understanding of the marking process. The findings revealed notable improvements across all areas, particularly in confidence and understanding of the OSCE format. A total of 12 students responded to both the pre- and post-intervention questionnaires, providing insights into the effects of the intervention.

The virtual reality group showed a statistically significant increase in mean confidence for OSCE scores from 2.83 (±0.41) to 3.83 (±0.41), with a mean difference of 1.00 (p=0.012). Additionally, there was a statistically significant increase in comfort with using VR technology from 2.33 (±1.51) to 4.00 (±0.63), with a mean difference of 1.67 (p=0.0545) (Table 1).

**Table 1:** Comparison of self-perceived confidence scores pre- and post-intervention for the virtual reality group Paired t-tests were used to compare pre- and post-intervention self-perceived scores for confidence, anxiety, knowledge of OSCE marking, knowledge of how OSCEs are run and comfort with virtual reality technology in the virtual reality group. Results are presented as mean (±SD). Mean differences, p-values and t-values are reported for each comparison. Statistical significance was set at p<0.05. SD: standard deviation, OSCE: Objective Structured Clinical Examination, VR: virtual reality

Category	Pre-intervention mean (SD)	Post-Intervention mean (SD)	Mean difference	t-value	p-value
Confidence for OSCEs	2.83 (±0.41)	3.83 (±0.41)	1.00	3.87	0.012
Anxiety regarding OSCEs	3.17 (±0.75)	2.83 (±0.98)	-0.33	-0.79	0.465
OSCE marking knowledge	2.17 (±0.75)	3.33 (±1.21)	1.17	2.15	0.084
Knowledge of how OSCEs are run	3.00 (±0.89)	3.50 (±0.55)	0.50	1.17	0.296
Comfort using VR technology	2.33 (±1.51)	4.00 (±0.63)	1.67	2.50	0.055

Other categories, such as "OSCE marking knowledge" (p=0.0842), showed notable improvements but did not reach statistical significance. Specifically, anxiety regarding OSCEs showed a reduction in mean anxiety from 3.17 (±0.75) to 2.83 (±0.98); however, this was not statistically significant.

There was also a statistically significant improvement in confidence for OSCEs in the control group with an improvement from 2.50 (±0.55) to 3.67 (±0.52), with a mean difference of 1.17 (p=0.0127) (Table 2).

**Table 2:** Comparison of self-perceived confidence scores pre- and post-intervention for the control group Paired t-tests were used to compare pre- and post-intervention self-perceived scores for confidence, anxiety, knowledge of OSCE marking, knowledge of how OSCEs are run and comfort with virtual reality technology in the control group. Results are presented as mean (±SD). Mean differences, p-values and t-values are reported for each comparison. Statistical significance was set at p<0.05. SD: standard deviation, OSCE: Objective Structured Clinical Examination, VR: virtual reality

Category	Pre-intervention mean (SD)	Post-intervention mean (SD)	Mean difference	t-value	p-value
Confidence for OSCEs	2.50 (±0.55)	3.67 (±0.52)	1.17	3.80	0.013
Anxiety regarding OSCEs	3.67 (±1.51)	3.00 (±1.41)	-0.67	-2.00	0.102
OSCE marking knowledge	3.00 (±0.89)	3.67 (±1.03)	0.67	1.58	0.175
Knowledge of how OSCEs are run	3.00 (±1.26)	4.00 (±0.63)	1.00	2.24	0.076
Comfort with using VR technology	2.00 (±0.89)	2.00 (±0.00)	0.00	0.00	1.000

Performance Global Rating Scales

The 16 students each underwent three OSCE stations. The marks for each station can be seen in the Appendices.

**Figure 1:**
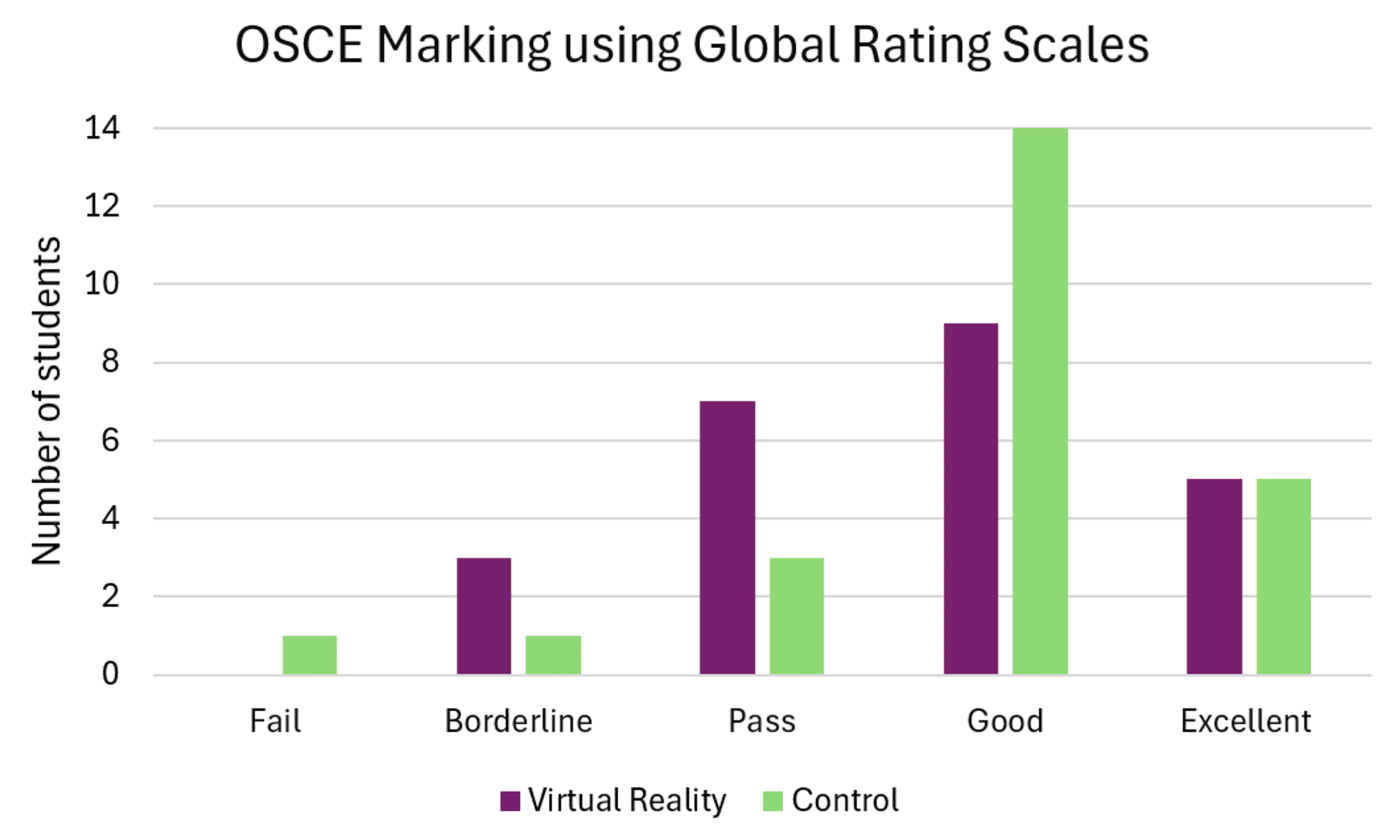
OSCE marking using Global Rating Scales OSCE: Objective Structured Clinical Examination

Out of 24 stations, 87.5% from the VR group and 91.7% from the control group were scored on the Global Rating Scale score as a pass or higher. There was no significant difference between the groups (X2=0.223, p=0.637). A greater percentage of students scored a good or above in the control group, as seen in Figure 1; however, this was not statistically significant (X=2.424, p=0.119).

Qualitative results

Focus Group

Overall, the focus group with the VR group feedback supports the potential benefits of 360-degree videos for OSCE preparation, particularly in terms of reducing anxiety and creating an immersive learning environment. They felt the use of VR offers the possibility of improving the diversity of patient cases in OSCE preparation resources. However, the limitations highlighted the need to adapt to VR before usage and ways to make it more accessible for neurodiverse students. This suggests that addressing these issues could enhance the learning experience and make the tool even more effective for OSCE preparation. Table 3 summarises these key themes with sub-themes and quotations.

**Table 3:** Summary of key themes with sub-themes and quotations identified from the virtual reality arm focus group VR: virtual reality, OSCE: Objective Structured Clinical Examination

Themes	Sub-themes	Supporting quotations
Immersive learning experience	Immersivity	"Helps prepare us for what it was actually like, so you can feel more comfortable." "I was like 'Oh, it really feels like I'm in the moment'."
	Realistic scenarios	"Just having this replica of the environment and personalised feedback … was really useful to mimic the actual exam environment."
Emotional well-being	Authentic preparation, increased confidence, reduced anxiety	"Now I definitely feel a bit more comfortable." "I feel like the VR has just alleviated some of it (stress)."
Understanding of the OSCE process	Real-time feedback in videos, realistic environment and feedback, integration of examiner comments, highlighting areas of improvement	"Seeing the feedback in real time was really useful." "It helps to … integrate the comment into context." "Seeing how they still get marks for making mistakes gave me a lot less stress."
	Familiarity with the OSCE marking process, clarifying OSCE examiner expectations	"I actually got more of an idea of going 'Oh, that's what they're (examiners) looking for,' rather than being given an acronym on a PowerPoint and being expected to, in some way, regurgitate that."
Diversity and inclusivity	Disease-specific diversity	"For certain disease processes that only affect or majorly affect certain ethnic groups."
	Promotes diversity	"If you're witnessing a lot more VR consultation, that means you're going to see a lot more diversity in patients."
Limitations of virtual reality	Adapting to new technologies, difficulty multitasking	"It was a bit sudden … I pressed play and was straight into the consultation. It would help to have a brief intro before starting the VR, giving me time to adjust." "Perhaps adding an audio element to it (for) … someone who's dyslexic." "As someone with dyslexia, it was difficult to read something in that frame of time."
	Technical difficulties	"The comments were behind me, so I had to twist my whole body."

We identified five major themes that emerged from the participants' feedback.

The first was the immersivity of the virtual learning environment, which was highlighted as a key strength of the simulation. Students overwhelmingly expressed that the virtual setting not only mimicked the actual examination environment but also mirrored the physical layout, making them feel as though they were genuinely in a real-life consultation. This level of realism, they noted, created a more authentic experience, allowing them to engage deeply with the material and develop a sense of preparedness for the real OSCEs.

The second and third major themes revolved around emotional well-being and understanding of the OSCE process. Many students emphasised how the virtual platform positively affected their confidence, reducing anxiety whilst improving their clarity on how OSCEs operate. Real-time feedback in the videos was another highly valued feature, with participants explaining that the immediate comments during simulated OSCE scenarios were instrumental in pinpointing areas for improvement and reinforcing successful techniques. This feedback, they felt, accelerated their learning process. Furthermore, students praised the transparency of the marking process, which helped to alleviate concerns about how OSCEs are assessed. This increased understanding gave them greater confidence in their ability to navigate the examination. The 360-degree videos were particularly impactful, providing students with a comprehensive view of the OSCE process. These videos allowed them to familiarise themselves with the examination environment in a way that traditional methods could not, giving them both a clearer mental picture and a heightened sense of readiness.

The penultimate theme was diversity and inclusivity. Students appreciated the potential for diversity and inclusivity in the 360-degree video cases, noting that the virtual format could represent a wider range of cases than is possible in real OSCEs due to limitations in the demographics of volunteer participants.

The final theme focused on the limitations of virtual reality technology. Students suggested a brief introductory period to help them acclimatise to the VR environment, making the transition smoother. They also recommended adding audio components to improve accessibility for students with specific learning needs. Concerns were raised about the placement of examiner comments in the 360-degree videos, with suggestions to adjust the positioning and allow the video to pause when comments appear for better engagement.

## Discussion

This study aimed to investigate the non-inferiority of 360-degree videos as a learning tool compared to traditional methods for pre-clinical medical students preparing for Objective Structured Clinical Examinations (OSCEs). Our findings highlight both the strengths and limitations of 360-degree videos, suggesting potential areas of improvement and integration with the medical school curriculum.

With respect to increasing student examination confidence, our study revealed the non-inferiority of VR compared with the live synchronous PowerPoint lecture received by the control group. Upon conducting a literature review, we found similar evidence of non-inferiority of VR compared to using traditional face-to-face teaching methods in simulated patient assessment scenarios [8].

There was a reduction in anxiety after using 360-degree videos in the post-intervention quantitative questionnaire; however, this result was not statistically significant. However, in the focus group, following the OSCEs, students reflected, and one of the main emerging themes was the reduction in anxiety. Many participants felt that the VR experience helped them become more comfortable with the OSCE process, alleviating some of the stress associated with the examination. Students appreciated both positive and constructive feedback on the simulated student's performance, as this was a real student and not an actor, the scenario was more authentic and thus allowed feedback to target common student pitfalls. This further clarifies how a simulated OSCE is marked and relieves student anxiety about high-stakes assessments such as OSCEs, which suggests that 360-degree videos may have a significant positive impact on student well-being and preparedness. Year one students in formative OSCEs at the University of Leicester also receive real-time formative feedback from their examiners, which is invaluable. VR could be a way for less-prepared or more anxious students to practice further.

We considered why there was a reduction in anxiety in the post-intervention quantitative questionnaire in the VR group, which was not statistically significant. We hypothesise that as students filled out the questionnaire immediately after using the 360-degree videos, there may have been an acute increase in anxiety due to the realisation that extensive preparation is needed for OSCEs, which was a new experience for all of them. This will have been their first exposure to the format of an OSCE and understanding how to apply all the skills they have been taught.

In the control group, there was also a reduction in anxiety; however, this was also not statistically significant and had a larger mean difference. This could be due to the teaching given during the traditional method and the familiarity of the format.

In addition, immersivity was a key theme identified from the focus group comments. The immersive environment of 360-degree videos helped create a low-pressure setting, allowing students to practise and become more comfortable with OSCE scenarios, thus alleviating some of the anxiety typically associated with OSCE preparation. This improved focus and attention during learning.

A study looking at the student experience with varying immersion levels of virtual reality simulation showed that students find the immersivity of the technology important and enjoy being able to move around and explore the environment [9]. Leicester Medical School was the first UK medical school to adopt a one-iPad-per-student programme at the undergraduate level to promote equitable access to online resources. We ensured that all of the 360-degree video resources were compatible with viewing from these university-provided devices.

Participants appreciated how the realistic, simulated OSCE environment demystified the process and enabled them to contextualise and apply theoretical knowledge more effectively than traditional methods. The inclusion of real-time embedded feedback was particularly valuable, as students welcomed the targeted examiner comments, which helped them adjust their performance during the OSCE. This interactive feedback loop was seen as especially beneficial for pre-clinical students, who often have limited access to real-world clinical scenarios before their OSCEs. This was reassuring as some studies have shown that immersive VR can be distracting for some students [10].

However, participants noted issues with adjusting to the VR environment. A study looking at the use of virtual reality in teaching skeletal anatomy also found similar issues with adjusting to the VR environment [11]. Some of our focus group comments were regarding improving the positioning of the examiner comments and recommended adding a short introduction to allow students to familiarise themselves with the VR environment before using the videos. In addition, students suggested ways to make the videos more accessible.

The Global Rating Scales showed non-inferiority of the VR group compared to the control group, although the control group received a 30-minute lecture and the VR group watched the videos right before the mock OSCE. There was no significant difference in passing grades between the two groups, demonstrating the non-inferiority of the VR intervention.

Perhaps in the future, with an increase in the use of virtual reality in the medical school curriculum, we will train faculty on how to utilise and incorporate this into their teaching. This will help develop the software and ensure educators are trained on maximising the benefits of VR educational resources.

Strengths and limitations

The exploratory design of our descriptive study provided a well-rounded evaluation by integrating quantitative data on 360-degree video use with qualitative insights into user perceptions. The qualitative nature of our methodology allows for greater flexibility regarding external validity concerns, making the sample size suitable for this preliminary study. This approach enabled us to gather detailed feedback on the tools' effectiveness and user experiences, offering valuable contextual insights.

However, our study design had weaknesses as well. The generalisability of the findings is constrained by several factors, including volunteer participation, a small sample size and recruitment from a single institution. Although there was strong interest from students and all engaged with the OSCE resources and mock OSCE, four students did not complete the post-course questionnaire. As a result, their data could not be included in the quantitative analysis. Another study could consider a larger sample size or integration of VR resources into their preparation for OSCEs.

Future directions

Given our ongoing involvement in projects exploring the use of virtual reality in medical education, this study adds to the growing body of evidence supporting the potential of VR as a valuable educational tool. In the focus group, students discussed how useful they found integrating the VR resources before the mock OSCE; perhaps this is something we can investigate in a future study. Although time constraints prevented us from including classroom-based practice before the mock OSCE, in the future, we can consider incorporating the classroom-based practice in the control group, as well as a similar intervention in the intervention group, such as a chatbot, to allow for individual practice with a simulated patient. Ideally, we would aim for a larger sample size from multiple institutions in future studies.

## Conclusions

The qualitative and quantitative results from this study align to demonstrate the non-inferiority of 360-degree videos compared with live synchronous teaching as an effective tool for pre-clinical medical students preparing for OSCEs. The immersive nature of these videos improved students' preparedness, confidence and understanding of the examination format by providing real-time feedback, compared to their pre-intervention baseline. Although the impact on anxiety was mixed, familiarity with the OSCE process helped to reduce stress.

In terms of non-inferiority, 360-degree videos and VR offer similar benefits to face-to-face teaching whilst promoting focus and active learning, although it was challenging for some students to adjust to. In the future, we aim to harness the power of these technologies even further to allow greater accessibility and to promote diversity in medical education. Whilst improvements can be made in optimising the VR experience and broadening access, these findings support the integration of immersive technologies into medical education.

## Appendices

Pre-questionnaire form

The pre-questionnaire form is shown in Table 4. The purpose of this questionnaire is to gather information about the VR group's prior experience with OSCEs and familiarity with virtual reality technology. This information will help us to better understand the impact of our educational resources on medical students' preparedness for OSCEs. All information was kept anonymous.

**Table 4:** Pre-questionnaire form OSCE: Objective Structured Clinical Examination

Question number	Question	Response options
1	Please select a random 5 digit code with numbers and letters. Write this down for future use.	5-digit code input
2	Which group are you in (traditional or virtual reality)?	Group selection (traditional/virtual reality)
3	How confident do you feel about your ability to perform well in an OSCE?	1: not at all confident, 5: extremely confident
4	How anxious are you about OSCEs?	1: not at all anxious, 5: extremely anxious
5	How would you rate your knowledge of the marking process of OSCEs?	1: not knowledgeable at all, 5: extremely knowledgeable
6	How would you rate your knowledge of how OSCEs are run on the day?	1: not knowledgeable at all, 5: extremely knowledgeable
7	How comfortable are you with using virtual reality technology?	1: not at all comfortable, 5: extremely comfortable

Post-questionnaire form

The post-questionnaire form is shown in Table 5. The purpose of this questionnaire is to gather feedback on the educational resources the VR group received as part of this study and to assess the impact of these resources on their preparedness for OSCEs. Their honest feedback will help us to improve these resources and better support them in preparing for OSCEs. All feedback was kept anonymous.

**Table 5:** Post-questionnaire form OSCE: Objective Structured Clinical Examination

Question number	Question	Response options
1	Please enter the same 5 digit code that you created when completing the PRE-questionnaire.	5-digit code input
2	Which group are you in (traditional or virtual reality)?	Group selection (traditional/virtual reality)
3	How confident do you feel about your ability to perform well in an OSCE?	1: not at all confident, 5: extremely confident
4	How anxious are you about OSCEs?	1: not at all anxious, 5: extremely anxious
5	How would you rate your knowledge of the marking process of OSCEs?	1: not knowledgeable at all, 5: extremely knowledgeable
6	How would you rate your knowledge of how OSCEs are run on the day?	1: not knowledgeable at all, 5: extremely knowledgeable
7	How comfortable are you with using virtual reality technology?	1: not at all comfortable, 5: extremely comfortable

Performance Global Rating Scales

The marks of the participants for each station are shown in Table 6.

**Table 6:** Global Rating Scale OSCE marking OSCE: Objective Structured Clinical Examination

	Participant	History-taking station 1	History-taking station 2	Triadic consultation
Virtual reality group	1	Good	Good	Pass
	2	Pass	Good	Borderline
	3	Good	Excellent	Pass
	4	Good	Pass	Borderline
	5	Excellent	Excellent	Good
	6	Good	Good	Excellent
	7	Borderline	Pass	Pass
	8	Excellent	Good	Pass
Control group	9	Good	Good	Excellent
	10	Pass	Good	Pass
	11	Good	Good	Fail
	12	Excellent	Excellent	Good
	13	Excellent	Good	Good
	14	Good	Pass	Good
	15	Good	Good	Good
	16	Excellent	Good	Borderline
